# Developmental genoarchitectonics as a key tool to interpret the mature anatomy of the chondrichthyan hypothalamus according to the prosomeric model

**DOI:** 10.3389/fnana.2022.901451

**Published:** 2022-08-04

**Authors:** Gabriel N. Santos-Durán, Susana Ferreiro-Galve, Sylvie Mazan, Ramón Anadón, Isabel Rodríguez-Moldes, Eva Candal

**Affiliations:** ^1^Grupo NEURODEVO, Departamento de Bioloxía Funcional, Universidade de Santiago de Compostela, Santiago, Spain; ^2^CNRS-UMR 7232, Sorbonne Universités, UPMC Univ Paris 06, Observatoire Océanologique, Paris, France

**Keywords:** catshark, *Scyliorhinus canicula*, segmental, *Otp*, *Dlx*, Pax6, *Pitx2*, 5-HT

## Abstract

The hypothalamus is a key vertebrate brain region involved in survival and physiological functions. Understanding hypothalamic organization and evolution is important to deciphering many aspects of vertebrate biology. Recent comparative studies based on gene expression patterns have proposed the existence of hypothalamic histogenetic domains (paraventricular, TPa/PPa; subparaventricular, TSPa/PSPa; tuberal, Tu/RTu; perimamillary, PM/PRM; and mamillary, MM/RM), revealing conserved evolutionary trends. To shed light on the functional relevance of these histogenetic domains, this work aims to interpret the location of developed cell groups according to the prosomeric model in the hypothalamus of the catshark *Scyliorhinus canicula*, a representative of Chondrichthyans (the sister group of Osteichthyes, at the base of the gnathostome lineage). To this end, we review in detail the expression patterns of *ScOtp*, *ScDlx2*, and *ScPitx2*, as well as Pax6-immunoreactivity in embryos at stage 32, when the morphology of the adult catshark hypothalamus is already organized. We also propose homologies with mammals when possible. This study provides a comprehensive tool to better understand previous and novel data on hypothalamic development and evolution.

## Introduction

The terminology used by columnar neuroanatomists to name forebrain regions and cell groups was largely based on the brain model originally postulated by Herrick (1910), which assumes the existence of longitudinal functional units or columns along the entire brain and supposes that such columns follow a straight longitudinal axis parallel to that of the body that extends from the hindbrain to the telencephalon. Herrick’s (1910) columnar model of vertebrate forebrain organization relied on *adult* morphological data as a basis for establishing homologies among territories in different vertebrates. Based on adult ventricular sulci and neglecting the ontogenic cephalic flexure existing in all vertebrate brains, this model divided the diencephalon into four longitudinal dorso-ventrally arranged columns as follows: epithalamus, pars dorsalis thalami, pars ventralis thalami, and hypothalamus. Kuhlenbeck (1929) also used ventricular sulci to establish homologies and divided the diencephalon into horizontal (longitudinal) units. For descriptive convenience and using morphological traits, Le Gros Clark (1938) divided the hypothalamus into three “from before backward” regions, designated supraoptic (also known as anterior), tuberal, and mamillary. Cytoarchitectonic and topographical criteria were used by this author to define the main nuclei within hypothalamic regions in the adult hypothalamus.

However, when studying early forebrain *development* in different vertebrates, the use of similar morphological criteria offered a different perspective. Haller von Hallerstein (1929) also considered the ventricular sulci as a basis for the homologation of forebrain organization across species but believed that sulci are related to embryonic transverse neuromeres, which imply a vertical rather than a horizontal subdivision of the diencephalon (consequently, the almost horizontal aspect of these sulci within this region was explained by the rotation of the embryonic diencepahlon at the cephalic flexure). Instead of relying on sulci, Bergquist (1932) and Kallén (1951) held that nuclear structure (i.e., cytoarchitecture), as seen in the embryonic brain, was primarily important to homologize areas of the brain. Pioneering embryological studies from these authors (Bergquist and Källén, 1954) suggested that diencephalic subdivisions were direct derivations of transversally oriented embryonic neuromeres. Interestingly, these authors suggested that the hypothalamus was divided into two transverse bands, which continued into the telencephalon.

The prosomeric model (Puelles and Rubenstein, 1993, 2003, 2015; Puelles et al., 2012) was postulated in 1993 under the influence of the 19th-century neuromeric brain models and was further developed and updated since then (for a review see Puelles, 2021). At its core, the model recognizes that the longitudinal axis of the forebrain curves following the cephalic flexure, which implies that the hypothalamus is topologically anterior or rostral to the diencephalon. This perspective implies that transverse segments of the forebrain are topologically oriented perpendicular to the longitudinal axis of the brain and recognized the acroterminal domain as its transverse rostral end (Puelles et al., 2012). The model relies on morphological data (i.e., relative position within the *Bauplan*) to establish homologies among vertebrates and provides a causal explanation to observed developmental gene expression patterns, experimental fate mapping, and functional analyses, among other lines of evidence. According to this view, the hypothalamus is not included in the diencephalon proper (which is divided into p3, p2, and p1 transverse segments or prosomeres) but in the secondary prosencephalon, which includes the telencephalon (dorsal) and the hypothalamus (ventral). The hypothalamo-diencephalic boundary (HDB) separates the diencephalon from the secondary prosencephalon. The intrahypothalamic boundary (IHB) divides both the telencephalon and the hypothalamus into two prosomeres: caudal or peduncular (hp1) and rostral or terminal (hp2). In the telencephalon, the IHB sets the boundary between the evaginated (peduncular) telencephalon, which includes the pallium and part of the subpallium, and the unevaginated (terminal) telencephalon occupied by the subpallial preoptic area. In the hypothalamus, the IHB also sets the boundary between peduncular and terminal subdivisions. Finally, the acroterminal region, being the rostralmost part of the neural tube, hosts unpaired telencephalic and hypothalamic structures (Puelles et al., 2012; Puelles and Rubenstein, 2015; Díaz and Puelles, 2020).

The use of gene expression patterns to identify subdivisions of the developing central nervous system, coined neural genoarchitecture or genoarchitectonics (Puelles and Ferran, 2012) or genetic neuroanatomy (Joyner and Sudarov, 2012), has been a key approach to identify homolog hypothalamic domains in different vertebrates (Puelles et al., 2000; Wullimann and Rink, 2001; Puelles and Medina, 2002; Osório et al., 2005, 2010; Del Giacco et al., 2006; Herget et al., 2014; Affaticati et al., 2015; Domínguez et al., 2015; Herget and Ryu, 2015; Santos-Durán et al., 2015, 2016, 2018; Albuixech-Crespo et al., 2017; Moreno et al., 2018; Schredelseker and Driever, 2020; López et al., 2022). However, changes in gene expression patterns occurring during early mid-development complicate the direct identification of these domains in the adult. This, together with the fact that the terminology conventionally used in neuroanatomy textbooks to refer to the adult brain follows columnar assumptions, hinders progress toward finding terminological or conceptual correspondences between molecularly defined embryonic territories and their derivatives in the adult brain.

Addressing these questions in chondrichthyans (cartilaginous fishes) is of utmost importance because of their phylogenetic position at the base of the gnathostome lineage as the sister group of bony fishes/land vertebrates (osteichthyans), which allows identifying lineage-specific diversifications and inferring ancestral states fixed prior to the gnathostome radiation (Carrera et al., 2008a,^2012^; Quintana-Urzainqui et al., 2012, 2015; Pose-Méndez et al., 2014, 2016; Hernández-Núñez et al., 2021). Much knowledge about the catshark brain neuroanatomy came from studies in adults describing the brain in columnar terms. The reference works of Smeets et al. (1983) and Smeets (1998), largely used as reference atlases for adult Chondrichthyans brains, interpreted the hypothalamus *sensu lato* of adult cartilaginous fishes in columnar and functional terms, and divided it into two main territories based on sulcal pattern, cell masses and fiber connections: the preoptic area, belonging morphologically to the caudal part of the unevaginated impaired telencephalon but functionally related to the hypothalamo-hypophyseal system, and the hypothalamus (hypothalamus *sensu stricto*) belonging to the ventral region of the diencephalon, located under the ventral thalamus, from which it is separated by the sulcus thalamo-hypothalamicus.

Recent developmental studies in chondrichthyans support the prosomeric model of forebrain organization, including the bending of the alar-basal boundary around the cephalic flexure and the rostral location of the hypothalamus relative to this axis. Several histogenetic domains have been recently identified in the hypothalamus of catshark embryos (*Scyliorhinus canicula*, a representative of cartilaginous fishes) by analyzing *ScOtp*, *ScDlx2*, *ScPitx2* gene expression, and Pax6-immunoreactivity, among other transcription factors (Santos-Durán et al., 2015, 2016, 2018). These markers, in combination or alone, were useful for identifying hypothalamic boundaries and five dorso-ventrally arranged histogenetic domains with their corresponding peduncular and terminal segmental subdivisions. However, the correspondence of the histogenetic domains defined in early embryos with their derivatives in the adult remains, to date, largely unexplored.

An attempt to establish terminological or conceptual correspondences between the defined brain territories according to the prosomeric model in the juvenile catshark and the adult brain territories named according to the reference work of Smeets et al. (1983) was previously proposed by Sobrido-Cameán et al. (2020) in their study of the expression of five prosomatostatin genes, but developmental gene expression patterns typically used to identify embryonic histogenetic domains in the hypothalamus were not considered in this study. Here, we present a comprehensive comparison between the morphologic interpretation of relevant gene expression data interpreted according to the prosomeric model in embryos at stage 32 (when the basic adult brain organization becomes appreciable) and the location of functional neuronal cell groups defined in adults (Smeets et al., 1983, and subsequent works following columnar terms). This work provides a framework to clarify the catshark hypothalamus nomenclature under a modern comparative neuromorphological view, which may be a useful tool for whoever addresses new research on cartilaginous fishes.

## Materials and methods

### Experimental animals

Embryos of the catshark (also named lesser-spotted dogfish; *S. canicula* L.) were supplied by the Marine Biological Model Supply Service of the CNRS UPMC Roscoff Biological Station (France). Additional embryos were kindly provided by Aquaria of Gijón (Asturias, Spain), O Grove (Pontevedra, Spain), and Finisterrae (A Coruña, Spain). Embryos were staged by their external features according to Ballard et al. (1993). For more information about the relationship of embryonic stages with body size, gestation, and birth, see Table 1 in Ferreiro-Galve et al. (2010). Ten stage-32 embryos were used in this study. Eggs from different broods were raised in seawater tanks in standard conditions of temperature (15–16°C), pH (7.5–8.5), and salinity (35 g/L). Adequate measures were taken to minimize animal pain or discomfort. All procedures conformed to the guidelines established by the European Communities Council Directive of 22nd of September 2010 (2010/63/UE) and by the Spanish Royal Decree 1386/2018 for animal experimentation and were approved by the Ethics Committee of the University of Santiago de Compostela.

### Tissue processing

Embryos were deeply anesthetized with. 5% tricaine methane sulfonate (MS-222; Sigma, St. Louis, MO, United States) in seawater and separated from the yolk before fixation in 4% paraformaldehyde (PFA) in elasmobranch’s phosphate buffer [EPB:0.1 M phosphate buffer (PB) containing 1.75% urea, pH 7.4] for 48-72 h depending on the stage of development. Subsequently, they were rinsed in phosphate-buffered saline (PBS), cryoprotected with 30% sucrose in PB, embedded in OCT compound (Tissue Tek, Torrance, CA), and frozen with liquid-nitrogen-cooled isopentane. Parallel series of sections (12–20 μm thick) were cut in transverse and sagittal planes on a cryostat and mounted on Superfrost Plus (Menzel-Glasser, Madison, WI, United States) slides.

### Immunohistochemistry on sections

For heat-induced epitope retrieval, sections were pre-treated with.01 M citrate buffer (pH 6.0) for 30 min at 95°C and allowed to cool for 20–30 min at room temperature (RT). Sections were then rinsed twice in 0.05 M Tris-buffered saline (TBS; pH 7.4) for 5 min each and incubated overnight with the primary antibody (rabbit anti-Pax6 polyclonal antiserum, Covance, Emeryville, CA, United States diluted 1:400; monoclonal mouse anti-proliferating cell nuclear antigen [PCNA] Sigma, St Louis, MO, diluted 1:500; rabbit anti-serotonin [5-HT] polyclonal antiserum, DiaSorin, Immunostar, Hudson, WI, United States, diluted 1:5000). Appropriate secondary antibodies (horseradish peroxidase [HRP]-conjugated goat anti-rabbit and anti-mouse, Biorad, diluted 1:200) were incubated for 2 h at RT. All dilutions were made with TBS containing 15% donkey normal serum (DNS; Millipore, Billerica, MA, United States), 0.2% Triton X-100 (Sigma), and 2% bovine serum albumin (BSA, Sigma), and incubations were carried out in a humid chamber. Then, sections were rinsed three times in TBS for 10 min each and the immune complex was developed with 0.25 mg/ml diaminobenzidine tetrahydrochloride (DAB, Sigma) and 0.00075% H_2_O_2_ in TBS pH 7.4, or with SIGMAFAST™ 3.3-DAB tablets as indicated by the manufacturers. Sections were rinsed in distilled water (twice for 30 min), allowed to dry for 2 h at 37°C, and mounted in Mowiol 4-88 Reagent (Calbiochem, Merk KGaA, Darmstadt, Germany).

### Controls and specificity of the antibodies

No immunostaining was detected when primary or secondary antibodies were omitted during incubations. Controls and specificity of the anti-Pax6 antibody in catshark brain were performed as described in Ferreiro-Galve et al. (2012). The monoclonal anti-PCNA antibody specifically labels proliferating cells in the brain, retina, and olfactory epithelium of this species (Rodríguez-Moldes et al., 2008; Ferrando et al., 2010; Ferreiro-Galve et al., 2010; Quintana-Urzainqui et al., 2015). Controls of specificity of the anti-5-HT antiserum in the catshark brain were previously performed in Carrera et al. (2008b).

### *In situ* hybridization on sections

We applied *in situ* hybridization for *ScOtp* (Santos-Durán et al., 2015, 2016, 2018), *ScDlx2* (Quintana-Urzainqui et al., 2012; Compagnucci et al., 2013; Debiais-Thibaud et al., 2013; Santos-Durán et al., 2015, 2016, 2018), and *ScPitx2* (Lagadec et al., 2015; Santos-Durán et al., 2018) genes in stage-32 brain sections using standard protocols. These probes were selected from a collection of *S. canicula* embryonic cDNA libraries (mixed stages S9 to S22), constructed in pSPORT1, and submitted to high-throughput EST sequencing. Selected cDNA fragments were cloned in pSPORT vectors. Sense and antisense digoxigenin-UTP-labeled and fluorescein-UTP-labeled probes were synthesized directly by *in vitro* transcription using them as templates for linearized recombinant plasmid DNA or cDNA fragments prepared by PCR amplification of the recombinant plasmids. Briefly, sections were permeabilized with proteinase K, hybridized with sense or antisense probes overnight at 65°C, and incubated with the alkaline phosphatase-coupled anti-digoxigenin or anti-fluorescein antibodies (1:2000, Roche Applied Science, Manheim, Germany) overnight at 4°C. The color reaction was performed in the presence of BM-Purple (Roche). Control sense probes did not produce any detectable signal.

### Image acquisition and analysis

Bright field images were obtained with an Olympus BX51 photomicroscope equipped with an Olympus DP71 color digital camera. Fluorescent sections were photographed with an epifluorescence photomicroscope Olympus AX70 fitted with an Olympus DP70 color digital camera. Photographs were adjusted for brightness and contrast and plates were prepared using Adobe Photoshop CS4 (Adobe, San Jose, CA, United States).

## Results

**FIGURE 1:**
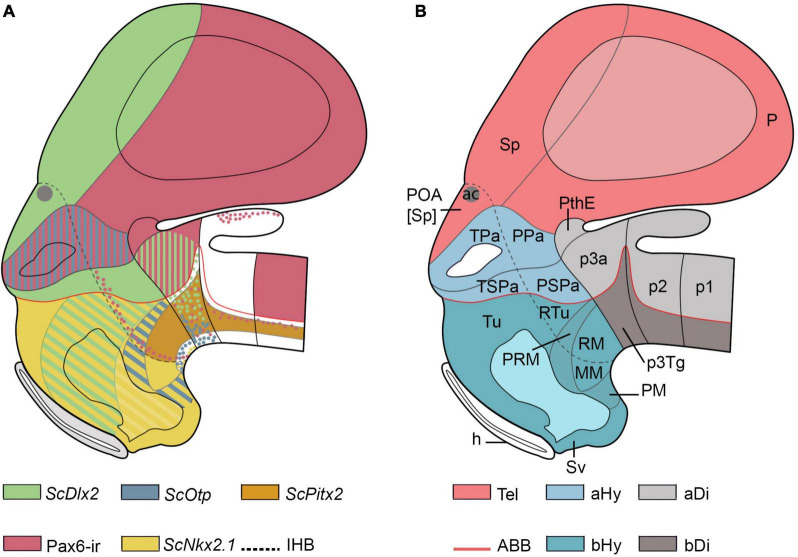
Schematic representations of the forebrain organization in *Scyliorhinus canicula* embryos at stage 29 according to previous genetic neuroanatomical data following the prosomeric model. **(A)** Schematic representation of *ScOtp-*, *ScDlx2-*, *ScNkx2.1-*, *ScPitx2*-expression and Pax6-ir structures in the secondary prosencephalon and surrounding tissues according to Santos-Durán et al. (2015, 2016, 2018). **(B)** Schematic representation of the segmental organization of the prosencephalon and main histogenetic domains of the secondary prosencephalon according to Santos-Durán et al. (2015, 2016, 2018). The boundary of the acroterminal region was not represented, but this region includes the pre-optic area, the pre-optic recess, optic stalk and eye vesicle, the optic chiasma, the tuberal median eminence, the acroterminal part of the infundibulum, and the neurohypophysis and ends at the mamillary body. (For abbreviations, see list).

Five hypothalamic histogenetic domains were previously identified by us in the hypothalamus of early catshark embryos by analyzing *ScOtp*, *ScDlx2*, and *ScPitx2* gene expressions and Pax6-immunoreactivity patterns (Figure 1A; stage 29 was used for representations since, at this stage, all studied markers exhibit a strong expression and sharp domain boundaries; see Santos-Durán et al., 2015, 2016, 2018). Following the prosomeric model, these territories were identified as terminal and peduncular paraventricular region (TPa/PPa; *ScOtp*-expressing and Pax6-ir domain), terminal and peduncular subparaventricular region (TSPa/PSPa; *ScDlx2* expressing domain), tuberal and retrotuberal region (Tu/RTu; *ScDlx2*-expressing domain), perimamillary and periretromamillary region (PM/PRM; *ScOtp*-expressing domain), and mamillary and retromamillary region (MM/RM; only the RM expresses *ScPitx2*; Figures 1A,B). The IHB separating terminal and peduncular subdivisions were either identified based on the course of serotonin (5-HT)-immunoreactive fibers in the medial forebrain bundle (mfb) or by the distribution of Pax6-immunoreactive (-ir) cells (Santos-Durán et al., 2015, 2016, 2018).

The catshark hypothalamus is limited dorsally with the telencephalon, which includes: the preoptic area (POA), a non-evaginated telencephalic subpallial compartment derived from hp2 located between the anterior commissure and the TPa/PPa hypothalamic domain (the *ScOtp*-expressing region of the alar hypothalamus; Figures 1A,B; Santos-Durán et al., 2015, 2018; Rodríguez-Moldes et al., 2017); and the evaginated telencephalon, derived from hp1. The hypothalamus is caudally limited with the rostral diencephalic prosomere (p3), formed in its alar part by the prethalamus (p3a) and its dorsal extension, the prethalamic eminence (PThE), and in its basal part by the tegmentum corresponding to this segment (p3Tg).

To relate the embryonic hypothalamic domains (identified by genetic anatomical data in the catshark *S. canicula* according to the prosomeric model) with their derivatives (developed functional nuclear groups), we analyzed in detail the expression patterns of *ScOtp*, *ScDlx2*, and *ScPitx2*, as well as Pax6-immunoreactivity at stage 32.

### *ScOtp* expression and 5-HT-immunoreactivity

The *ScOtp* expression has been described in the developing catshark embryo (Santos-Durán et al., 2015, 2016, 2018), but it has not been analyzed in detail at stage 32 when the basic morphology of the adult brain of *S. canicula* becomes recognizable. Here we study the expression pattern of *ScOtp* as a relevant marker for several prosomeric domains, in comparison with the distribution of 5-HT-immunoreactive (-ir) cell bodies and fibers found in characteristic structures of the chondrichthyan hypothalamus (see Carrera et al., 2008b), termed the paraventricular organ (PVO) and the posterior recess organ (PRO), following the nomenclature of Meurling and Rodríguez (1990).

**FIGURE 2:**
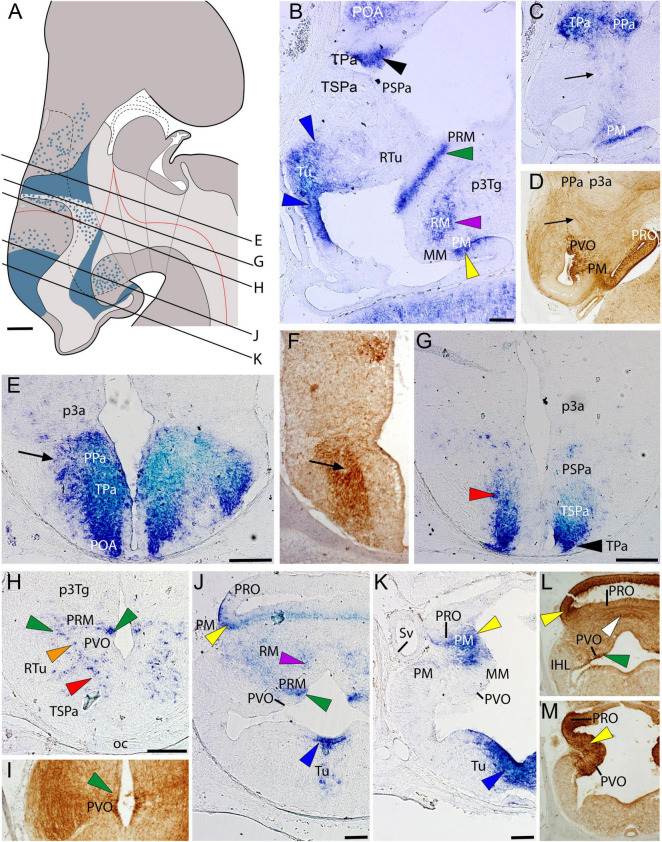
Regionalization of the hypothalamus of *Scyliorhinus canicula* at stage 32 based on the expression of *ScOtp* (blue color in **A**) by means of *in situ* hybridization **(B,C,E,G,H,J,K)** and immunohistochemistry for 5-HT **(D,F,I,L,M)** in sagittal **(B–D)** or transverse **(E–M)** sections. Scheme on **(A)** represents a maximal projection of the expression found in sagittal and parasagittal sections. Lines represent the level of transverse sections. Ventricular labeling is represented with solid color while mantle cells are represented with dots. Black arrowheads point expression in the TPa. Blue arrowheads point expression in the Tu. Green arrowheads point expression in the PRM. Yellow arrowheads point expression in the PM. Purple arrowheads point expression in the RM. Red arrowheads point *ScOtp*-expressing cells in TSPa. Orange arrowhead points *ScOtp*-expressing cells in RTu. Arrows in **(C,D)** point the tracts of the medial forebrain bundle. For abbreviations, see list. Scale bars: 125 μm.

At stage 32, *ScOtp* presents an expression pattern similar to that observed in previous developmental stages (Figures 2A–C,E,G,H,J,K). Many *ScOtp*-expressing cells can be found in subpallial territories adjacent to the TPa/PPa (Figures 2A,B,E). In the alar hypothalamus, *ScOtp* is broadly expressed in the mantle zone of the TPa/PPa domain. Scattered *ScOtp*-expressing cells can be also observed in the ventricular zone (Figures 2A–C). In the rostralmost region of the TPa domain, *ScOtp* is expressed in the walls of what was termed the preoptic recess by Smeets et al. (1983) (PR; black arrowheads in Figures 2B,G). Ventral to this recess, *ScOtp*-expressing cells are observed in the mantle zone of the TSPa/PSPa (red arrowhead in Figures 2G,H). In the basal hypothalamus, *ScOtp* is expressed in the ventricular and mantle zones of the rostralmost Tu domain (blue arrowheads in Figures 2B,J,K) but also in the RTu mantle zone (orange arrow in Figure 2H). *ScOtp* has also been detected in the ventricular and mantle zones of the PRM domain (green arrowheads in Figures 2B,H,J). It is worth noting that *ScOtp*-expressing cells of the ventralmost TSPa/PSPa domain are continuous with those of the RTu and PRM domains (red, orange, and green arrowheads in Figure 2H). *ScOtp*-expression is also detected in cells of the PM domain (yellow arrowheads in Figures 2B,J,K) and in the RM domain (purple arrowheads in Figures 2B,J).

Concerning 5-HT-immunoreactivity at stage 32 (see also Carrera et al., 2008b), immunopositive fibers coursing in the mfb are observed ascending to the telencephalon through the alar hypothalamus (compare arrows in Figures 2C–F). More ventrally, the 5-HT-ir cells of the PVO (Figure 2I) are recognized abutting *ScOtp* expression in the PRM ventricular domain (compare green arrowheads in Figures 2H,I). 5-HT-immunoreactivity is also recognized abutting *ScOtp* expression in the rostralmost PRM (compare PVO and green arrows in Figures 2J,L). The PVO is continuous with the PRO, both comprising CSF-contacting neurons and fibers immunoreactive for 5-HT (Figure 2M). Moreover, *ScOtp* expression in more rostral positions of the PM domain co-distributes with part of the 5-HT-ir PRO (compare yellow arrowheads in Figures 2J–M). The 5-HT-ir fibers are abundantly detected crossing the floor plate of the RM domain (white arrow in Figure 2L; see also Santos-Durán et al., 2015).

### *ScDlx2* expression

The expression of *ScDlx2* has been analyzed in detail in the catshark subpallium at late embryonic stages (Quintana-Urzainqui et al., 2012, 2015). In the hypothalamus, to date, the expression of *ScDlx2* has been only analyzed in the early and middle embryonic stages (Santos-Durán et al., 2015, 2016, 2018).

**FIGURE 3:**
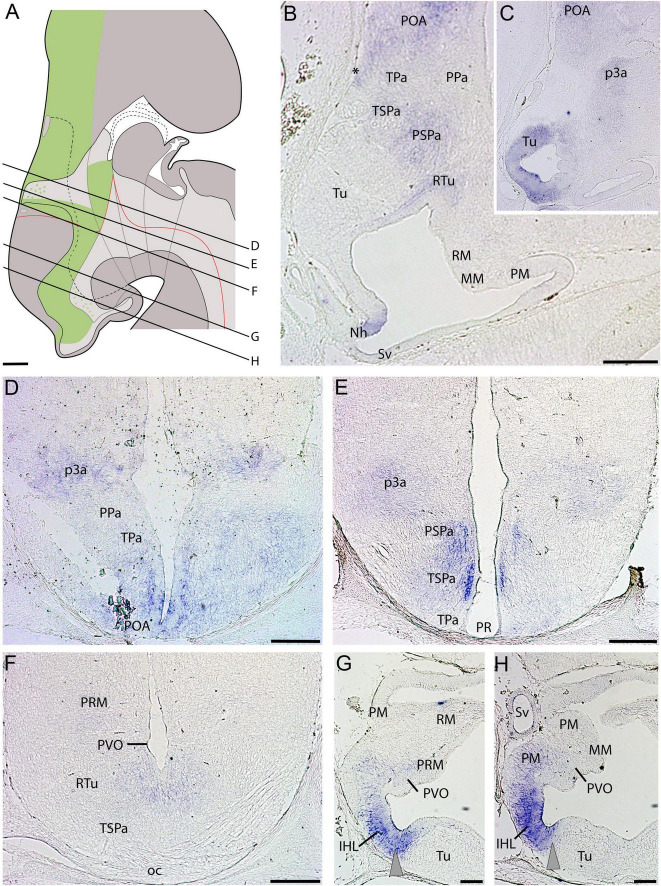
Regionalization of the hypothalamus of *Scyliorhinus canicula* at stage 32 based on the expression of *ScDlx2* (green color in **A**) by means of *in situ* hybridization (**B–H**) in sagittal (**B,C**) or transverse (**D–H**) sections. Scheme on **(A)** represent a maximal projection of the expression found in sagittal and parasagittal sections. Lines represent the level of transverse sections. Ventricular labeling is represented with solid color while mantle cells are represented with dots. Gray arrowheads in **(G,H)** point to *ScDlx2* expression in the inferior hypothalamic lobes. For abbreviations, see list. Scale bars: 125 μm.

At stage 32, *ScDlx2* expression is similar to that already described at earlier developmental stages (Figures 3A–H). In the alar plate, the *ScDlx2* signal is recognized in the rostral and ventralmost subpallium dorsal to the TPa/PPa domain (POA in Figures 3B,D). *ScDlx2* is continuously expressed in the TSPa/PSPa domain of the alar hypothalamus (Figures 3B,E,F), and the alar plate (prethalamus) of prosomere p3 (Figures 3C–E). Note that the TSPa domain is associated to withe optic chiasm (oc in Figure 3F). In the basal hypothalamus at stage 32, two *ScDlx2*-expressing domains can be detected inside the Tu/RTu domain (Figures 3B,C,F–H). A domain of intense *ScDlx2* expression is coextensive with the inferior hypothalamic lobes (IHL in Figures 3G,H), which corresponds to the Tu. There is a less intense domain spreading caudalwards from the IHL to the PVO (Figures 3G,H) that corresponds to the RTu domain (Figure 3F). It is noteworthy that expression can be detected in the neurohypophysis (Nh) at this stage (Figure 3B).

### Pax6 immunoreactivity

Pax6-immunoreactivity has been previously analyzed in the diencephalon and secondary prosencephalon of early and late catshark embryos (Ferreiro-Galve et al., 2008; Ferreiro Galve, 2010). The organization of the hypothalamus under the updated prosomeric framework (Puelles et al., 2012; Puelles and Rubenstein, 2015) has been recently addressed at early embryonic stages (Santos-Durán et al., 2016) but not from stage 32 onward.

**FIGURE 4:**
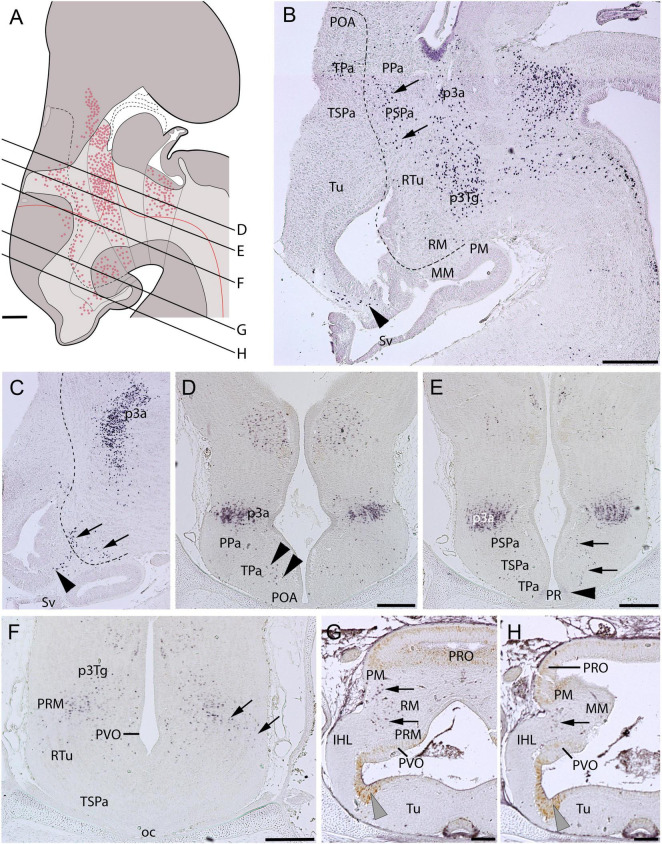
Regionalization of the hypothalamus of *Scyliorhinus canicula* at stage 32 based on immunohistochemistry against Pax6 (red color in **A**; **B–F**) and PCNA (**G,H**) in sagittal (**B,C**) or transverse (**D–H**) sections. Scheme on **(A)** represent a maximal projection of the expression found in sagittal and parasagittal sections. Lines represent the level of transverse sections. Ventricular labeling is represented with solid color while mantle cells are represented with dots. Dashed lines in **(B,C)** represent the IHB. Arrows in **(B,C)** point to Pax6-ir cells following the IHB. Black arrowheads in **(C)** point to Pax6-ir cells in the Tu. Black arrowheads in **(D)** show Pax6-ir cells at the POA (subpallium)/TPa border. Black arrowhead in **(E)** point to Pax6-immunoreactivity in the PR. Gray arrowheads in **(G,H)** point to PCNA-immunoreactivity in the inferior hypothalamic lobes. For abbreviations, see list. Scale bars: 125 μm.

At stage-32 embryos, Pax6-ir cells are detected in the alar and basal plates of the hypothalamus (Figures 4A–F). In the alar hypothalamus, Pax6-immunoreactivity is detected at the limit between the TPa and the subpallium (arrowheads in Figure 4D; compare with Figure 3D). Some faintly labeled cells are observed in the ventricular lateral walls of the TPa domain, at the level of the PR (arrowhead in Figure 4E). Pax6-ir cells spread dorso-ventrally in the mantle of the TPa down to the RM domain following roughly the IHB (see dashed lines in Figures 4B,C, and arrows in Figures 4B,C,E,F; see also Santos-Durán et al., 2016). Scattered Pax6-ir cells spreading in the mantle zone can be detected in the Tu domain rostral to the IHB (black arrowheads in Figures 4B,C). Finally, in the rostralmost diencephalon Pax6-ir cells are observed in p3, both in alar (p3a; Figures 4B–E) and basal (p3Tg; Figures 4B,F) regions. PCNA-ir cells are observed in the ventricular zone of the IHL (Figures 4G,H). Scarce PCNA-ir cells can be observed in the PVO and PRO (Figures 4G,H).

### *ScPitx2* expression

**FIGURE 5:**
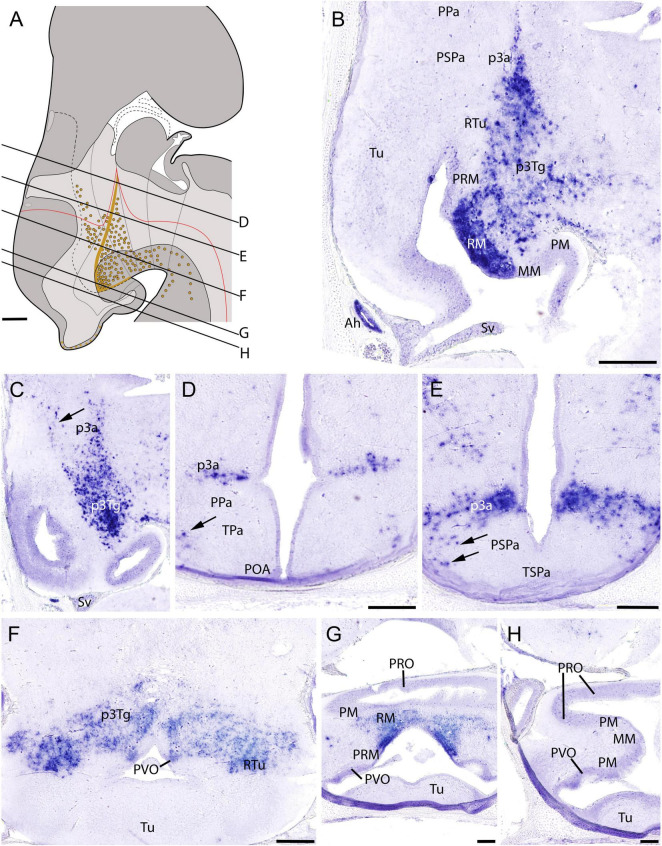
Regionalization of the hypothalamus of *Scyliorhinus canicula* at stage 32 based on the expression of *ScPitx2* (orange color in **A**) by means of *in situ* hybridization (**B–H**) in sagittal (**B,C**) or transverse (**D–H**) sections. Scheme on **(A)** represent a maximal projection of the expression found in sagittal and parasagittal sections. Lines represent the level of transverse sections. Ventricular labeling is represented with solid color while mantle cells are represented with dots. For abbreviations, see list. Scale bars: 125 μm.

The expression of *ScPitx2* has been analyzed in early embryos by Lagadec et al. (2015) and Santos-Durán et al. (2018), but not in the context of the mature hypothalamic organization. At stage 32, as in former stages of development, *ScPitx2*-expressing cells are abundant and continuously observed in the basal plate starting from the RM domain and extending beyond it, in the zona limitans intrathalamica (zli) and the alar (p3a) and basal (p3Tg) region of the rostralmost diencephalon (Figures 5A–H). In the basal region, *ScPitx2* expression is not observed rostral to the RM domain (Figures 5B,C,F–H). It is noteworthy that scattered *ScPitx2*-expressing cells are observed in the alar peduncular hypothalamus, roughly following the IHB (Figures 5A–C, arrows in Figures 5D,E). *ScPitx2* is also expressed in dispersed cells of the early Sv (Figures 5B,C) and the adenohypophysis (Figure 5B).

## Discussion

The hypothalamus is a key physiologic center of the vertebrate brain, involved in individual (i.e., water intake, feeding, and thermoregulation) and species-specific (i.e., reproduction and aggression) survival responses (Butler and Hodos, 2005). Variations in hypothalamic structure and/or function throughout evolution are probably related to biological diversity in vertebrates in these responses. Molecular approaches for the identification of the relative position of different hypothalamic histogenetic domains of the catshark within the *Bauplan*, conforming to the updated prosomeric model, have been key to establishing meaningful homologies with other vertebrates (Santos-Durán et al., 2015, 2016, 2018).

**FIGURE 6:**
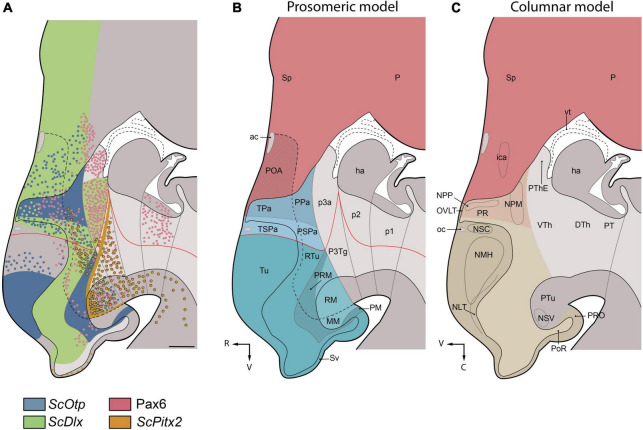
Parasagittal schematic representation of *Scyliorhinus canicula* hypothalamus at stage 32 based on the expression patterns of developmental genes **(A)** and comparison of the regionalization of the *S. canicula* hypothalamus according to prosomeric **(B)** and columnar **(C)** frameworks. For reference, the ventricle line has been drawn at midline (sagittal) level. **(A)** Maximal projection of the expression of *ScOtp* (blue) *ScDlx2* (green), Pax6 (red) and *ScPitx2 (orange)* found in sagittal and parasagittal sections **(B)** Prosomeric subdivisions in the hypothalamus and surrounding tissues. Telencephalon is represented in red; different domains of the hypothalamus are represented in different shades of blue. The red line represents the postulated alar-basal boundary. According to this model, the hypothalamus excludes the POA, lies ventral (V) to the telencephalon and rostral (R) to the diencephalon. **(C)** Hypothalamus, according to the columnar model and approximate location of main cell masses as described in the adult shark by Smeets et al. (1983). Telencephalon is represented in red; hypothalamus is represented in brown. According to the columnar model, the hypothalamus is located caudal (C) to the telencephalon and is conceived as the ventral (V) part of the diencephalon. Note that NPP and NPM (red shaded area) were considered in Smeets et al. (1983) as belonging morphologically to the telencephalon, but they were described together with other hypothalamic cell masses because of their functional relationship to the hypothalamohypohyseal system. For abbreviations, see list.

**FIGURE 7:**
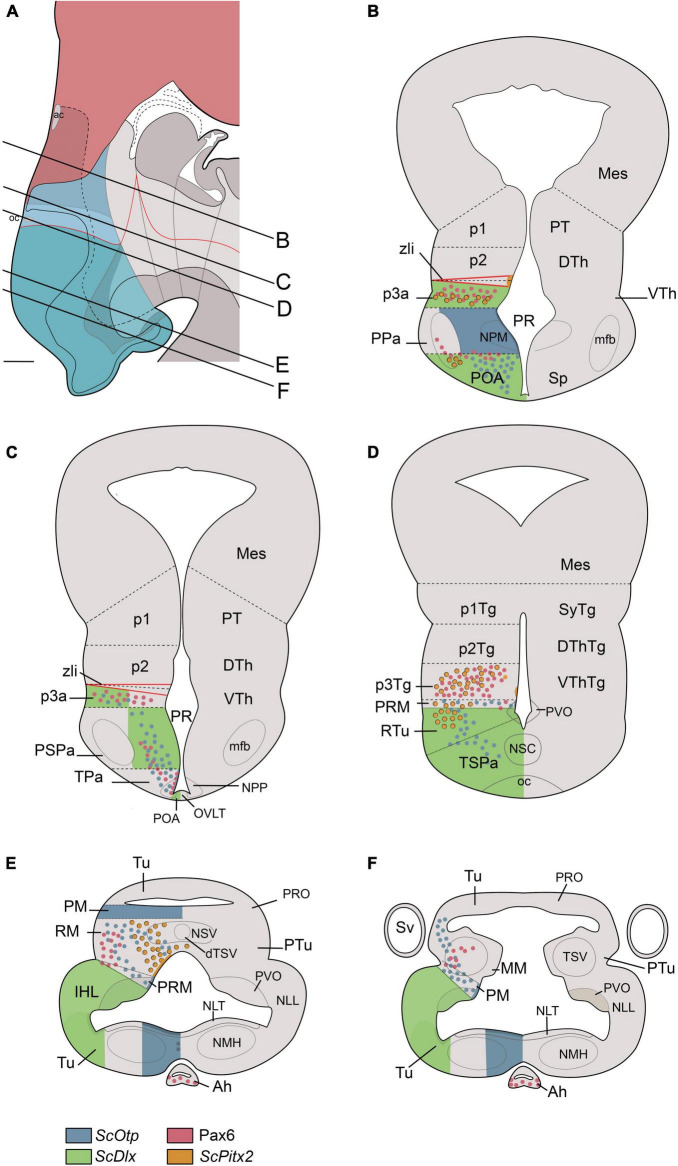
Transverse schematic representations of *Scyliorhinus canicula* hypothalamus at stage 32 according to prosomeric (left) and columnar (right) frameworks. Lines in **(A)** represent the level of transverse sections in **(B–F)**. Territories in **(A)** are colored as in Figure 6B. For abbreviations, see list.

**FIGURE 8:**
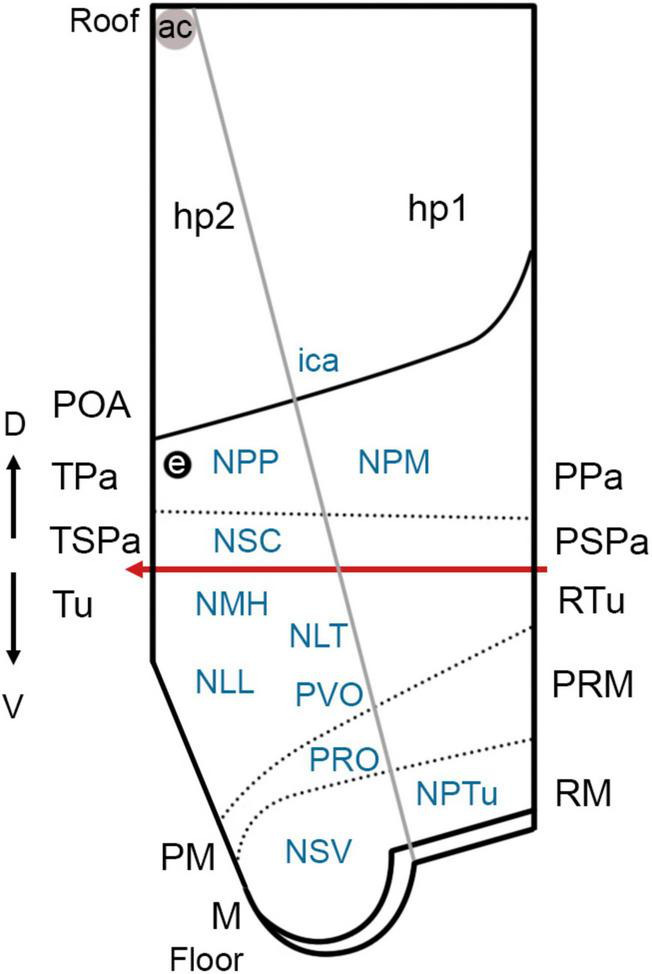
Simplified view of the prosomeric model (only the secondary prosencephalon is represented) in *Scyliorhinus canicula* (based on the schematic representation of the updated prosomeric model by Puelles and Rubenstein, 2015) and main cell masses harbored in each hypothalamic domain. The red line represents the alar-basal boundary (rostral is to the left). For abbreviations, see list.

We discuss here the terminological and conceptual correspondences between embryonic hypothalamic territories (identified at stage 32 by differential gene expression patterns that support the prosomeric model; Figures 6A,B) and developed nuclear groups derived from these territories (which had been described in the reference atlas of Smeets et al. (1983), following columnar assumptions; Figures 6C, 7, 8).

### Telencephalo-hypothalamic boundary

The boundary between the telencephalon and alar hypothalamus in sharks has been identified by the adjacent expression of *ScDlx2* in the subpallium and *ScOtp* in the dorsal part of the alar hypothalamus (TPa/PPa; Figures 2A, 3A, 6A,B, 7; Santos-Durán et al., 2015, 2016). *Dlx2-* and *Otp-*expressing domains occupying the same topological positions have been described in mice, birds, reptiles, amphibians, and zebrafish (see Morales et al., 2021 and references therein). Interestingly, in mice, a domain within this *Otp*-expressing territory has been found to co-express *Foxg1*, a transcription factor expressed in the telencephalon during early development and often considered a good telencephalic marker (e.g., Manuel et al., 2010). This territory, where *Foxg1/Otp* show overlapping expression, was defined in embryos and postnatal mice and sauropsids as a distinct domain located at the transition between telencephalon and hypothalamus and termed *telencephalon-opto-hypothalamic domain* (TOH; Morales et al., 2021; Metwalli et al., 2022). While *ScFoxg1* and *ScOtp* expression patterns have been studied by us during early development in sharks (Santos-Durán et al., 2015), the absence of data from the same developmental stages prevents us from knowing whether there is an overlapping region of *ScFoxg1/ScOtp*-expression at the transition between the catshark telencephalon and hypothalamus.

In adolescent rats, Bilbao et al. (2022) have recently suggested that a spike-like caudal part of the TOH extending into the amygdala could correspond to what they have defined as the *extended preoptic area domain* (an extension of the preoptic area into the hp1 prosomere containing tyrosine hydroxylase [TH] positive cells) or to what was defined in mouse embryos as the *hypothalamo-amygdalar corridor* (a corridor of hypothalamic neurons that invade parts of the pallial amygdala, conceived as an evaginated part of the hypothalamic paraventricular area; García-Calero et al., 2021). In sharks, a corridor of Pax6-ir cells was observed from the peduncular part of the paraventricular hypothalamus (PPa, in hp1) to the telencephalon, which topologically could correspond to the *extended preoptic area* of rats. Whether this corridor invades what has been identified as the putative pallial amygdala of sharks (which also contain TH-positive cells; Rodríguez-Moldes et al., 2022) deserves further investigation.

### Alar hypothalamus

The alar hypothalamus comprises the TPa/PPa *ScOtp*-expressing and the TSPa/PSPa *ScDlx-2* expressing domains, which are dorso-ventrally arranged (Figures 2A, 3A, 6A,B). Two domains occupying equivalent topological positions have been found in the zebrafish forebrain, which respectively expresses *Otp* and *Dlx2* (Affaticati et al., 2015). It is noteworthy that, based on the location and shape of the forebrain ventricles, and considering the centrifugal gradient of progenitor cell maturation around them, these territories were not considered by Affaticati et al. (2015) as the alar hypothalamus but rather as belonging to a distinct morphogenetic unit, the optic recess region (ORR), which is distinct from the telencephalon and hypothalamus and from which the eye cup arose (Affaticati et al., 2015; Yamamoto et al., 2017). It must be noted that the ORR, which was previously termed the preoptic area in the mature zebrafish, corresponds to the region located between the anterior and the postoptic commissure and, therefore, comprises the prosomeric preoptic area and the prosomeric alar hypothalamus together (Affaticati et al., 2015). Similar analyses of dynamics of cell maturation around forebrain ventricles have not been performed in catshark, which precludes further comparisons.

#### Paraventricular hypothalamus (TPa/PPa)

In Chondrichthyans, the paraventricular hypothalamus (TPa/PPa) has been identified as a domain expressing *ScOtp* bounded ventrally by the *ScDlx2*-expressing domain of the subparaventricular hypothalamus (TSPa/PSPa) and caudally by the *ScDlx2*-expressing domain of p3a (present results; Santos-Durán et al., 2015, 2016).

Roughly speaking, the *ScOtp*-expressing TPa/PPa domain develops as the hypothalamic region in which well-conserved vasotocin-like populations can be found in the adult (see Vallarino et al., 1990a). Since vasotocin is produced from a prohormone that, after post-translational modifications, also yields the carrier protein neurophysin (e.g., Rodriguez-Santiago et al., 2017), these populations must have belonged to the neurophysin-positive nucleus reported by Meurling et al. (1996), which was termed magnocellular preoptic nucleus. This nucleus, together with its projections to the hypothalamus–hypophyseal tract and the neurointermediate lobe of the hypophysis, form the classic neurosecretory system. Oxytocin-like peptides (asvatocin and phasvatocin) isolated from the *S. canicula* pituitary (Chauvet et al., 1994) are likely to be expressed in cells of this domain, although this has not been assessed. It is noteworthy that the paraventricular hypothalamus (TPa/PPa) is probably the progenitor domain of other peptidergic cells found in adjacent territories (e.g., the subparaventricular hypothalamus; see below), given that *Otp* is involved in the development of such phenotypes in the hypothalamus in different vertebrates (Simeone et al., 1994; Acampora et al., 1999, 2000; Wang and Lufkin, 2000; Del Giacco et al., 2006; Bardet et al., 2008; Morales-Delgado et al., 2011, 2014; Puelles et al., 2012; Affaticati et al., 2015; Díaz et al., 2015), and that many migratory pathways of peptidergic neuronal populations have been described from the paraventricular hypothalamus toward neighboring regions (García-Moreno et al., 2010; Morales-Delgado et al., 2011, 2014; Puelles et al., 2012; Díaz et al., 2015). Indeed, we cannot discard that some peptidergic cells observed in the basal hypothalamus or diencephalon in catshark originated from the TPa/PPa since extensive migrations of neurons from the TPa/PPa to these territories are observed in mammals (Morales-Delgado et al., 2011, 2014; Puelles et al., 2012; Díaz et al., 2015). The TPa/PPa domain also seems to present a conserved glutamatergic neuronal phenotype (Villar-Cerviño et al., 2011; Puelles et al., 2012), which has not been studied in Chondrichthyans.

Of note, in sharks, *ScLhx5* is distinctly expressed in the TPa (but not in the PPa), supporting the existence of terminal and peduncular paraventricular subdomains. The boundary between TPa and PPa is further supported by the course of the 5-HT-ir fibers in the mfb and/or Pax6-ir cells in the PPa, the latter being the only useful landmark for the IHB at late stages (Figure 6; see also Santos-Durán et al., 2015, 2016). Moreover, two subdivisions of what had been termed the pre-optic nucleus (i.e., the parvicellular and magnocellular preoptic nuclei in Smeets et al., 1983) can be distinguished in the paraventricular domain since they present different peptides and different spatial distribution, which supports the existence of TPa and PPa subdivisions (Figures 6–8). Considering these nuclei are not preoptic (telencephalic) but paraventricular (hypothalamic), they should be renamed as *parvicellular and magnocellular paraventricular nuclei* (NPP; NPM). In mammals, rostrocaudal subdivisions can be identified by combinations of various molecular markers (Morales-Delgado et al., 2011, 2014; Puelles et al., 2012; Díaz et al., 2015; Ferran et al., 2015) and *Otp*-expressing cell clusters can be grouped into rostral and caudal (terminal and peduncular) paraventricular domains (Simeone et al., 1994; Acampora et al., 1999, 2000; Wang and Lufkin, 2000; Del Giacco et al., 2006; Bardet et al., 2008; Puelles et al., 2012).

#### Subparaventricular hypothalamus (TSPa/PSPa)

The *ScDlx2*-expressing TSPa/PSPa is located ventrally to the *ScOtp*-expressing TPa/PPa (Figures 2, 3, 6–8). In both chondrichthyans and mammals, this domain is also characterized by a GABAergic neuronal phenotype associated with the expression of *Dlx* genes (Carrera et al., 2008a; Ferreiro-Galve et al., 2008; Puelles et al., 2012; Santos-Durán, 2015; Santos-Durán et al., 2015, 2016). The TSPa/PSPa domain contains *ScOtp*-expressing cells probably migrated from the TPa/PPa (present results). These cells could give rise to different peptidergic cell types that have been described near the catshark optic chiasm (Met-Enkephalin, Vallarino et al., 1994; Tyrotropin Releasing Hormone, Teijido et al., 2002; Somatostatin, Sobrido-Cameán et al., 2020). Of note, Growth Hormone releasing hormone (Ghrh)-positive cells described in the PSPa of mammals have presumably migrated from the PPa (Morales-Delgado et al., 2014).

Terminal (TSPa) and peduncular (PSPa) subdivisions were identified in sharks in early developmental stages (up to stage 31) by the differential presence of Pax-6 ir cells in the mantle of PSPa (Santos-Durán et al., 2016), though a scarce number of Pax6-ir cells has been observed to invade TSPa at later stages (e.g., Figures 4A,E).

In sharks, the terminal subdivision, the TSPa, harbors the suprachiasmatic nucleus (NSC), an unpaired GABAergic and catecholaminergic cell group located caudal and ventral to the optic chiasm (Carrera de Figueiredo, 2008; Carrera et al., 2012). Considering its topological location and neurochemical markers, this nucleus could be equivalent to the suprachiasmatic and the anterior hypothalamic nuclei of mammals together (being the former free of TH-ir cells, and the latter corresponding to the ventral part of the catecholaminergic neuronal group identified alphanumerically as A14; Bilbao et al., 2022). In mammals, the suprachiasmatic nucleus receives projections from the retina and is involved in pacemaker circadian functions (Puelles et al., 2012; Gotlieb et al., 2020; Kim and Blackshaw, 2020). In Chondrichthyans comparable retinal connections have been described in the NSC (Smeets et al., 1983), suggesting similar functions. Dispersed *ScOtp*-expressing cells found here can explain the peptidergic and/or catecholaminergic cellular phenotypes of this nucleus (Vallarino et al., 1994; Teijido et al., 2002; Carrera et al., 2005, 2012; Sobrido-Cameán et al., 2020). Of note, catshark NSC catecholaminergic cells have homolog counterparts in mammals and may have been involved in neuroendocrine function by sending projections to the neurohypophysis (Carrera et al., 2012).

Finally, the entopeduncular nucleus of mammals, which originates from PPa (*Otp*) and PSPa (*Dlx*) subdivisions (Puelles et al., 2012), presents dispersed Pax6-positive cells (Stoykova et al., 1996). In Chondrichthyans, similar Pax6-ir cells can be recognized in the PSPa (Figure 4), suggesting homology with those described in mammals (see also Santos-Durán et al., 2016). Of note, in chondrichthyans, the nucleus named entopeduncular nucleus (Smeets et al., 1983) does not belong to the hypothalamus and, therefore, is not homologous to the nucleus of the same name in tetrapods.

### Basal hypothalamus

The catshark basal hypothalamus comprises three dorso-ventrally arranged domains (Tu/RTu, PM/PRM, and MM/RM) that can be identified by the combined expression of *ScOtp*, *ScDlx2*, *ScNkx2.1*, and *ScPitx2* (see also Santos-Durán et al., 2015, 2018). The basal hypothalamus harbors the territories referred to in Smeets et al. (1983) as the hypothalamus (*sensu stricto*) and the tuberculum posterioris (posterior tubercle, PTu; Figure 6C), both described in this book and atlas as diencephalic structures according to the columnar model.

#### Tuberal hypothalamus (Tu/RTu)

The Tu/RTu is mainly, but not exclusively, characterized by the expression of *Dlx* genes and cells with a GABAergic phenotype in both chondrichthyans and mammals (Carrera de Figueiredo, 2008; Puelles et al., 2012; Santos-Durán, 2015; Santos-Durán et al., 2015, 2018). The Tu/RTu comprises territories extending from the optic chiasm to the ventral end of the Sv (Figures 6, 7). The chondrichthyan Tu/RTu harbors several unpaired and paired structures with specific dorso-ventral locations, which, considered together with the differential expression of various molecular markers, support further dorso-ventral and rostrocaudal subdivisions, as with mammals (Morales-Delgado et al., 2011, 2014; Puelles et al., 2012; Díaz et al., 2015; Ferran et al., 2015).

The dorsalmost cell group in the catshark tuberal hypothalamus is the hypothalamic nucleus medius (NMH; Figures 6, 7D–F, 8). The territory that harbors the NMH expresses *ScOtp* (but not *ScDlx2*) and is coextensive with the region of the adenohypophysis expressing *ScPitx2* and Pax6 (data not shown). It has been described in adults as a rostrally (topologically dorsally) unpaired and caudally (topologically ventrally) paired nucleus that partially overlaps the median eminence, a well-known neurohemal organ (Smeets et al., 1983). The mammalian counterparts of these unpaired and paired portions could be the anterobasal nucleus and the arcuate nucleus, respectively. The mammalian anterobasal nucleus is an unpaired structure that expresses *Otp* at the early stages, although, later, these *Otp*-expressing cells migrate tangentially to the paired arcuate nucleus (Joly et al., 2007; Bardet et al., 2008; Morales-Delgado et al., 2011; Puelles et al., 2012). The arcuate nucleus consists of diverse neuroendocrine cell types and is tightly connected to the neurosecretory median eminence (Joly et al., 2007).

More ventrally in the Tu/RTu, we can find the median eminence, the neurointermediate lobe of the Nh (which maintains *ScDlx2*-expression as in former stages; Figures 3, 7, 8; see also Santos-Durán et al., 2015) and the Sv, an enigmatic circumventricular organ found in jawed fishes associated to circadian functions (Figures 6, 7E,F, 8; see also Sueiro et al., 2007; Nakane et al., 2013). The Sv is considered part of the Tu/RTu because it originates from the ventral portion of the infundibular walls (i.e., rostral- and ventral-most Tu; van de Kamer and Shuurmans, 1953; Sueiro et al., 2007) and presents dispersed *ScDlx2*- (Santos-Durán et al., 2015, 2018) and ScPitx2-expressing cells (present results). In mammals, it has been speculated that a thin underdeveloped territory of the rostralmost Tu could be homologous to the Sv since it presents a similar topology and ependymal character (Puelles et al., 2012). However, the ventral part of the Tu/RTu in mammals has been recently proposed as the hypothalamic ventricular organ, a linear longitudinal ependymal specialization of suggested organizer properties (Díaz and Puelles, 2020). Worth to note that at stage 32, the Sv presents abundant proliferating cells (PCNA-ir; see Figure 3H in Santos-Durán et al., 2018) compared with other hypothalamic regions, supporting its large expansion at later stages (van de Kamer and Shuurmans, 1953; Sueiro et al., 2007).

In more ventral portions of the chondrichthyan Tu, we found paired structures defined by the lack or the presence of expression of *ScDlx2*, which harbor the lateral tuberal nucleus (NLT) and the nucleus of the lateral lobes (NLL) in the IHL, respectively (Figures 7E,F, 8). In mammals, the ventromedial and dorsomedial hypothalamic nuclei (the latter lying topologically ventral to the former; Puelles and Rubenstein, 2015) present similar expression patterns (*Dlx*-negative and *Dlx*-positive, respectively; Morales-Delgado et al., 2011, 2014; Puelles et al., 2012), suggesting homologies with chondrichthyans. It is noteworthy that, at stage 32 (present results), the NLT is negative for the markers considered here, although it is positive for *ScLhx5* at previous stages (Santos-Durán et al., 2018). Similarly, the mammalian ventromedial nucleus seems to be positive for *Lhx5* and abuts *Dlx*-expressing territories at early stages (Sheng et al., 1997; Abellán et al., 2010), but it is negative at later stages (Szabó et al., 2009; Heide et al., 2015; Miquelajáuregui et al., 2015). The catshark NLT presents several types of peptidergic cells (somatostatin, Sobrido-Cameán et al., 2020; met-enkephalin and leu-enkephalin, Vallarino et al., 1994; melanin-concentrating hormone, Vallarino et al., 1989a; atrial natriuretic factor, Vallarino et al., 1990b; delta sleep-inducing peptide, Vallarino et al., 1992; beta-endorphin, Vallarino et al., 1989b; galanin, Vallarino et al., 1991; neuropeptide Y, Vallarino et al., 1988; corticotropin-releasing factor, Vallarino et al., 1989c; adrenocorticotropic hormone, Vallarino and Ottonello, 1987, Vallarino et al., 1989b), and is thought to be associated with the hypophysis, as reported in actinopterygian fishes (reviewed in Butler and Hodos, 2005). The mammalian ventromedial hypothalamus is involved in feeding, fear, thermoregulation, and sexual activity (Harding and McGinnis, 2005; King, 2006; Silva et al., 2016).

The NLL is located in the inferior hypothalamic lobes (IHL), which are distinctive structures of the hypothalamus of chondrichthyans and actinopterygian fishes. In chondrichthyans, the IHL constitutes the largest part of the adult hypothalamus. They are involved in feeding and aggression-related behavioral responses (Demski, 1977) and have been considered a major relay center between the telencephalon and brainstem because of their widespread ascending and descending connections (Smeets and Boord, 1985). At stage 32, the IHL walls present an intense proliferative activity, although the differential density of proliferating cells along these walls suggests that different subdivisions could emerge late in development, as pointed out in studies in a teleost (Corujo and Anadón, 1990). Interestingly, at this stage, the distribution of proliferating cells matches that of ScDlx2-expressing cells (compare Figures 3G,H, 4G,H). Of note, parts of the IHL of chondrichthyans have been reported to regulate feeding behavior, while the Dlx2-expressing mammalian basal hypothalamus has been involved in feeding-dependent circadian rhythms, wakefulness rhythms, temperature regulation, and energy expenditure (Mieda et al., 2006; Shimogori et al., 2010; Zhao et al., 2017; Pulix and Plagge, 2020; Kim and Blackshaw, 2020), which support some kind of homology between these nuclei. Moreover, the NLL could also harbor counterparts of the mammalian lateral hypothalamus (LH) expressing hypocretins/orexins. These peptidergic cells, involved in feeding behavior and sleep-wake cycle, originated from progenitors in *Lhx9*-expressing RTu territories that migrate to the LH (Shimogori et al., 2010; Dalal et al., 2013; Díaz et al., 2015). Homolog *ScLhx9*-expressing domains have been described in the catshark (Santos-Durán et al., 2016) and preliminary data from our lab suggest the presence of cells migrating toward the IHL (data not shown). Despite data supporting the role of the IHL in the feeding behavior of sharks (Evan et al., 1976; Demski, 1977), recent studies in zebrafish point out that the IHL could rather be involved in sensory integration (Bloch et al., 2019). However, the teleost IHL has a more complex origin, receiving a substantial population of projection neurons originating from the midbrain that is hardly comparable to any shark population (Bloch et al., 2019).

The ventralmost portion of the catshark Tu/RTu is characterized by a low *ScDlx2*-expression and will give rise to the paired portion of a circumventricular organ known in *S. canicula* as paraventricular organ (PVO; Rodríguez-Moldes, 2011). The ventricular surface of this organ is characteristically folded and contains a high density of cerebrospinal fluid (CSF)-contacting neurons of catecholaminergic, serotoninergic, and peptidergic nature (see Rodriguez-Moldes and Anadon, 1987; Meurling and Rodríguez, 1990; Molist et al., 1993; Carrera et al., 2012; Sobrido-Cameán et al., 2020). In mammals, the ventralmost Tu/Rtu is also *Dlx*-positive (Morales-Delgado et al., 2011, 2014; Puelles et al., 2012). A homologous circumventricular organ has been described in the same region (i.e., hypothalamic ventricular organ), extending through its rostro-caudal extension from the HDB to the rostralmost Tu (Puelles et al., 2012), as it happens in the catshark. In mammals, this ventralmost Tu/RTu has also been described to contain a progenitor domain of hypothalamic histaminergic neurons (Puelles et al., 2012). Histaminergic neurons are only present at the transition between tuberal and mammillary hypothalamus (Puelles et al., 2012), whose most characteristic cell group is the tuberomammillary nucleus involved in promoting sleep/wake transitions (Kim and Blackshaw, 2020). Histaminergic cells have not been, so far, investigated in sharks despite the relevant role of histaminergic neurons in teleosts (Sundvik and Panula, 2012).

Finally, in mammals, the A13 catecholaminergic group has been ascribed to the dorsal and caudalmost part of the RTu (Puelles et al., 2012; Bilbao et al., 2022), while these cells seem to arise from *Dlx-* and *Pax6*-positive cells originated from prosomere 3 (Mastick and Andrews, 2001; Andrews et al., 2003; Puelles et al., 2012). The A13 equivalent, which was interpreted as a dorsomedial hypothalamic (topologically caudal) TH-ir group observed in sharks by Carrera et al. (2012), can be interpreted to belong to the RTu domain. The presence of Pax6-ir cells in the catshark RTu area suggests that their dopaminergic cell groups (Carrera et al., 2012) have the same origin.

#### Perimamillary hypothalamus (PM/PRM)

Ventral to the *Dlx*-expressing Tu/RTu, *Otp* expression defines the PM/PRM hypothalamus of both chondrichthyans and mammals (Morales-Delgado et al., 2011, 2014; Puelles et al., 2012; Santos-Durán et al., 2015, 2018). In chondrichthyans, *ScOtp* expression can be recognized in the territory where an unpaired circumventricular organ named posterior recess organ (Meurling and Rodríguez, 1990) develops (Figures 6, 7D–F). Of note, according to the prosomeric model, this organ is not the caudalmost structure of the hypothalamus, nor does it derive from the mammillary region, and, therefore, the use of terms such as *posterior recess* or *mamillary recess*, can be misleading. Accordingly, we suggest the name of perimamillary recess and/or perimamillary recess organ (PRO) to refer to this structure derived from the PM/PRM. In adult catshark, a functional and structural continuity has been described between the PVO and the PRO-based on the common expression of peptides and monoamines (Meurling and Rodríguez, 1990; Rodríguez-Moldes, 2011). Previous studies revealed that *ScEmx2* expression roughly corresponds to the 5-HT-ir neuron-bearing region of the PVO and PRO (see Santos-Durán et al., 2018). However, differential expression of *ScDlx2* and *ScOtp* in domains in which the PVO and PRO will develop, respectively, suggests that these organs have a different origin. Whether the differential expression of *ScDlx2* and *ScOtp* is related to the development of the CSF-contacting aminergic and peptidergic neurons of these circumventricular organs remains to be investigated.

In mammals, *Otp* expression has not been described in the HVO (the equivalent of the PVO of anamniotes) but rather ventrally to this organ, thus, defining the PM/PRM (Puelles et al., 2012). Like with other *Otp*-expressing domains, the PM/PRM seems to be a rich glutamatergic domain that gives rise to the classic dorsal premamillary nucleus (Puelles et al., 2012). This nucleus has been involved in fear behavior (Canteras et al., 2020).

#### Mamillary hypothalamus (MM/RM)

According to previous studies in mammals, markers expressed in both the MM and RM are not known, except for those related to the glutamatergic phenotype (Szabó et al., 2009; Puelles et al., 2012). The MM is characterized by *Nkx2.1*, *Emx2*, or *Lhx5* expression and the RM by *Shh*, *Foxa1*, or *Pitx2* expression, although the limit between both areas can also be recognized by the 5-HT-ir retro- or supramamillary commissure (Szabó et al., 2009; Puelles et al., 2012; Santos-Durán et al., 2015, 2018). Moreover, markers expressed in the MM are typically expressed in other domains of the *Nkx2.1*-expressing hypothalamus, while markers expressed in the *Pitx2*-expressing RM are typically continuously expressed in the basal diencephalic prosomeres, also known as rich glutamatergic domains (Puelles et al., 2012). These data also suggest a different histogenetic hypothalamic organization that has been discussed in previous studies (Santos-Durán et al., 2018). In mammals, the caudal limit of the RM can be identified by the expression of *Nr5a1* in the basal plate of prosomere 3 (p3Tg; Puelles et al., 2012). It is noteworthy that conserved *Dlx-* and/or *Pax6*-positive postmitotic cells in prosomere 3 from chondrichthyans to mammals could also be useful markers (Figures 4, 6; see also Stoykova et al., 1996; Mastick and Andrews, 2001; Andrews et al., 2003; Ferreiro-Galve et al., 2008; Santos-Durán et al., 2016).

Classical studies of chondrichthyans consider the posterior recess organ (now named PRO; see above) as a caudalmost part of the hypothalamus (Molist et al., 1993; Teijido et al., 2002; Rodríguez-Moldes, 2011; Anadón et al., 2013). The present observations support a subdivision of the posterior tubercle in a rostral part derived from the MM/RM and a caudal part derived from p3Tg (tegmentum or basal plate of prosomere 3) (Figure 6; see also Santos-Durán et al., 2015, 2018). In fishes, the posterior tubercle is known by harboring important catecholaminergic populations involved in sensory-motor control (revised in Carrera et al., 2012). Genes like *Shh*, *Otp*, *Neurogenin*, *Foxa*, and *Pitx* seem to be involved in their origin (reviewed in Vernier and Wullimann, 2009), most of them being detected in sharks (Santos-Durán et al., 2015, 2018). Based on the differential expression of *ScNkx2.1, ScShh*, and *ScPitx2*, the hypothalamic part of the posterior tuberculum could be divided into a rostral domain (the MM, expressing *ScNkx2.1* but not *ScShh* or *ScPitx2*) and a caudal domain (RM, expressing *ScShh* and *ScPitx2* but not *ScNkx2.1*), harboring the nucleus of the saccus vasculosus (NSV) and the nucleus of the posterior tuberculum (NPTu), respectively (present results; see also Santos-Durán et al., 2015).

The NSV, which receives projections from neurons of the Sv, has been described rostral to the postinfundibular commissure (Smeets, 1998), likely homolog of the retromamillary commissure (see also Santos-Durán et al., 2015). Since the Sv is a structure found exclusively in jawed fishes, an equivalent nucleus cannot be recognized in tetrapods. Therefore, a possible relation of the NSV with the classic medial and lateral mamillary nucleus harbored in the mammalian MM (Puelles et al., 2012) is difficult to sustain.

The NPTu is located caudally to the retromamillary commissure and presents peptidergic cells (Molist et al., 1992) likely migrated from the *ScOtp*-expressing PM/PRM. The NPTu has been described as rich in catecholamines and peptidergic neuron phenotypes involved in sensorimotor control also associated with the basal ganglia (Figures 6, 7D,E, 8; Molist et al., 1992; Carrera et al., 2012; Quintana-Urzainqui et al., 2012; Santos-Durán et al., 2015, 2018). In mammals, the classic subthalamic nucleus that is involved in similar functions also emerges from the RM and expresses *Pitx2* (Martin et al., 2004; Puelles et al., 2012). It is noteworthy that the mammalian subthalamic nucleus has been described as an RM derivative containing migrated *Pitx2*-expressing cells that are finally located in the RTu (Puelles et al., 2012). Strikingly, in the catshark, there is a *ScPitx2*-expressing population that fits with this description (Figures 6, 7D,E), whose homology needs to be further investigated. The mammalian RM also gives rise to the classical lateral, medial retromamillary nucleus besides the migrated parasubthalamic nucleus and lateral tuberal nucleus.

## Conclusion

The hypothalamus is a key vertebrate brain region involved in survival and physiological functions. In *S. canicula*, prosomeres in the secondary prosencephalon and subdomains within the alar and basal hypothalamus had been previously identified by means of genoarchitectonic analyses (Santos-Durán et al., 2015, 2016, 2018), which have been key to identifying the homolog of hypothalamic domains in different vertebrates. However, much knowledge about the catshark brain neuroanatomy came from studies in adults describing the brain under columnar terms, which hindered progress toward finding terminological and conceptual correspondences between molecularly defined embryonic territories and their derivatives in the adult brain. This work provides a comparative framework to clarify the catshark hypothalamus nomenclature under a modern neuromorphological view, offering a key tool for past and future comparative studies using cartilaginous fish to study brain development and evolution.

## Data availability statement

The original contributions presented in the study are included in the article/supplementary material, further inquiries can be directed to the corresponding author.

## Ethics statement

The animal study was reviewed and approved by Ethics Committee of the University of Santiago de Compostela.

## Author contributions

GS-D, IR-M, and EC conceived and devised the study. GS-D and SF-G acquired the data. SM provided the probes for *in situ* hybridization experiments. GS-D, IR-M, and EC analyzed the data and drafted the manuscript. EC prepared the final version with contributions from all authors. IR-M and EC obtained funding. All authors contributed to the article and approved the submitted version.

## Abbreviations

ABB: alar-basal boundary
ac: anterior commissure
Ah: adenohypophysis
aDi: alar diencephalon
aHy: alar hypothalamus
bDi: basal diencephalon
bHy: basal hypothalamus
C: caudal
D: dorsal
DTh: dorsal thalamus
DThTg: dorsal thalamus tegmentum
dTSV: decussation of the tractus of the saccus vasculosus
e: eye
h: hypophysis
ha: habenula
HDB: hypothalamodiencephalic boundary
hp1: caudal or peduncular hypothalamus
hp2: rostral or terminal hypothalamus
ica: interstitial nucleus of the anterior commissure
IHB: intrahypothalamic boundary
IHL: inferior hypothalamic lobes
Mes: mesencephalon
mfb: median forebrain bundle
MM: mamillary area
Nh: neurohypophysis
NLL: nucleus lobi lateralis
NLT: nucleus lateralis tuberis
NMH: nucleus medius hypothalamic
NPM: magnocellular preoptic nucleus
NPP: parvicellular preoptic nucleus
NPTu: nucleus of the posterior tubercle
NSC: suprachiasmatic nucleus
NSV: nucleus of saccus vasculosus
oc: optic chiasm
OVLT: vascular organ of lamina terminalis
P: pallium
p1: prosomere 1
p1Tg: tegmental part of prosomere 1p2, prosomere 2
p2Tg: tegmental part of prosomere 2
p3: prosomere 3
p3a: alar part of prosomere 3
p3Tg: tegmental part of prosomere 3
PM: perimamillary area
POA: preoptic area
PPa: peduncular paraventricular area
PR: preoptic recess
PRM: periretromamillary area
PoR: posterior recess
PRO: posterior recess organ
PSPa: peduncular subparaventricular area
PT: pretectum
PThE: prethalamic eminence
PTu: posterior tubercle
PVO: paraventricular organ
R: rostral
RM: retromamillary area
RTu: retrotuberal domain
Sp: subpallium
Sv: saccus vasculosus
SyTg: synencephalic tegmentum
Tel: telencephalon
TPa: terminal paraventricular area
TSPa: terminal subparaventricular area
TSV: tractus of the saccus vasculossus
Tu: tuberal domain
V: ventral
vt: *velum transversum*
VTh: prethalamus (ventral thalamus)
VThTg: prethalamus (ventral thalamus) tegmentum
zli: zona limitans intrathamica.
